# Appraisal of synthetic cationic Gemini surfactants as highly efficient inhibitors for carbon steel in the acidization of oil and gas wells: an experimental and computational approach

**DOI:** 10.1039/d2ra02603a

**Published:** 2022-06-08

**Authors:** M. Abdallah, M. A. Hegazy, H. Ahmed, Arej S. Al-Gorair, H. Hawsawi, M. Morad, F. Benhiba, I. Warad, A. Zarrouk

**Affiliations:** Chem. Depart., Faculty of Applied Sciences, Umm Al-Qura University Makkah Saudi Arabia metwally555@yahoo.com; Chem. Depart., Faculty of Sciences, Benha University Benha Egypt; Egyp. Petr. Res. Inst. (EPRI) Nasr City Cairo Egypt; Chem. Depart., College of Sciences, Princess Nourah Bint Abdulrahman University Riyadh Saudi Arabia; Tabuk Univ. Tabuk Saudi Arabia; Laboratory of Advanced Materials and Process Engineering, Faculty of Sciences, Ibn Tofail University BP 242 14000 Kenitra Morocco; Laboratory of Materials, Nanotechnology, and Environment, Faculty of Sciences, Mohammed V University in Rabat P. O. Box. 1014 Agdal-Rabat Morocco azarrouk@gmail.com; Department of Chemistry, An-Najah National University P. O. Box 7 Nablus Palestine; Faculty of Pharmacy, Arab American University P. O. Box 249 Jenin Palestine

## Abstract

New cationic Gemini surfactant (CGS) molecules were synthesized and investigated as anticorrosive materials for carbon steel (CS) in 1 M HCl solution by chemical, electrochemical and theoretical studies such as DFT and MDS approaches. The anticorrosion efficacy increased with the increase in the CGS concentration. It reached 95.66% at 5 × 10^−3^ M of the CGS molecule using PDP measurements. PDP studies confirm that the CGS molecule acts as a mixed inhibitor. The EIS outcomes were explained by an equivalent circuit in which a constant phase element (CPE) rather than a double-layer capacitance (*C*_dl_) was exploited to donate a more precise fit of the experimental outcomes. The CGS molecule follows the Langmuir isotherm as it is chemically adsorbed onto the surface of CS. To explore the kinetic and adsorption mechanisms, the thermodynamic characteristics of the activation and adsorption processes were assessed under the impact of temperature. Frontier molecular orbitals (FMOs) were achieved by the density functional theory (DFT) method. The study of interatomic interactions at the [CS (Fe(110))]/CGS level was discussed using molecular dynamics (MD) simulation.

## Abstract 2

New cationic Gemini surfactant (CGS) molecules were synthesized and investigated as anticorrosive materials for carbon steel (CS) in 1 M HCl solution by chemical, electrochemical and theoretical studies such as DFT and MDS approaches.

## Graphical abstract

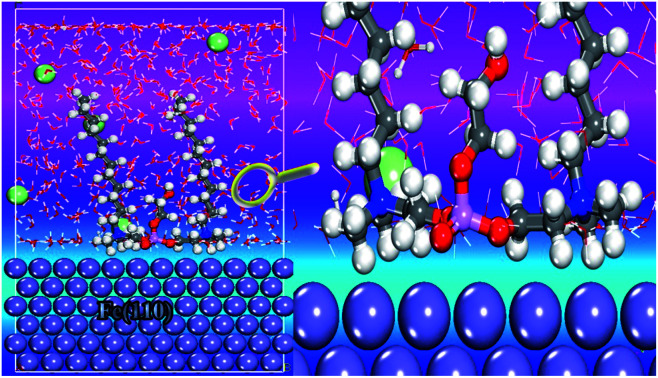

## 1 Introduction

The phenomenon of corrosion in the petroleum industry is widely studied by researchers because of its great importance to the national economy. The acidification of oil and gas wells by some acids such as hydrochloric acid is considered to dissolve the rocks that line the contours of the well, but unfortunately, the acid causes corrosion of the wells, and there are attempts by researchers in this field to solve this problem. Among these attempts to protect these tubular materials in the oil well from corrosion is the injection of suitable anti-corrosion compounds.^1^ Most common acid inhibitors are organic molecules including hetero atoms. The inhibition effectiveness of these compounds originates from its adsorption onto the surface of the steel constructing a covering film on the steel surface.^2–15^ This film retards the corrosion of the steel in the investigated acidic solution. The amount of adsorption is based on the kind and condition of the steel surface, the adsorption method, the chemical formula of the anticorrosion molecules, the type of corrosive solutions and other factors.^16,17^

Surfactants are organic molecules with unique characteristics due to their amphiphilic molecules. This is the reason for its unique application in the field of metal protection against corrosion. Surfactant inhibitors have several properties such as high anticorrosive efficacy, inexpensiveness, nontoxicity and easy preparation.^18,19^ Surfactants accumulate on the interfaces, thus controlling interactions between the substrate and its circumference, when added to the corrosive electrolyte in low amounts. Various types of surfactants have been mentioned to interpret the corrosion inhibitory effect, based on the surfactant ranking, substrate type, and anticorrosion dosage.^20,21^

The main objective of this work is to extend the scope of our previous work by exploiting new types of surfactants as corrosion inhibitors. These compounds are economically profitable because they are cheap, safe, not harmful to health and easy to prepare and give high inhibitory efficiency. In this work, we exploited a novel synthetic cationic Gemini surfactant (CGS) as an anticorrosive material of CS in 1 M HCl solution. Some chemical and electrochemical tests have been exploited to assess the inhibitory strength of the CGS molecule. The experimental study of these inhibitors must also be coupled with the theoretical investigation exploiting the DFT method and molecular dynamics “MD” simulation in order to get good visualization of the mechanism of action of CGS.

## 2 Experimental procedures

### 2.1 Synthesis of CGS molecules

**Scheme 1:**
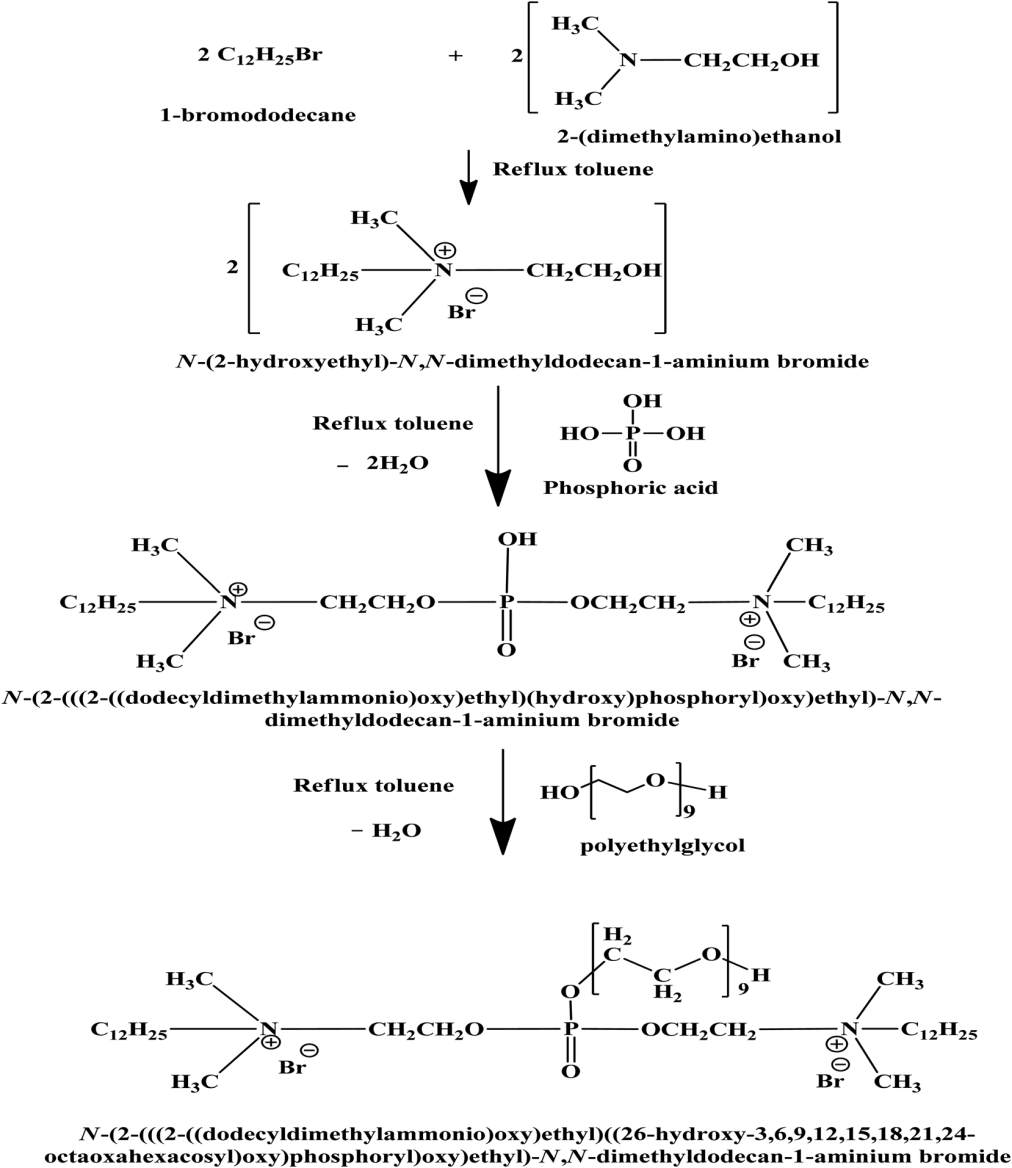
Preparation of the CGS molecule.

In this research, the CGS molecule was prepared *via* 3 steps, as illustrated previously.^13,17^ These steps produce the CGS molecule, namely, *N*-(2-(((2-((dodecyldimethylammonio)oxy)ethyl)((26-hydroxy-3,6,9,12,15,18,21,24-octaoxahexacosyl)oxy)phosphoryl)oxy)ethyl)-*N*,*N*-dimethyldodecan-1-aminium bromide. The method of synthesis of the CGS molecules is shown in Scheme 1. The CGS molecule was recognized by FTIR, ^1^HNMR, and ^31^PNMR spectroscopic analyses.

### 2.2 Carbon steel (CS)

The CS sheets or rods applied in this manuscript are produced by Iron and Steel Factory – Egypt.

The chemical composition of CS is (weight%) as follows: 0.192 carbon, 0.051 silicon, 0.940 manganese, 0.008 phosphorus, 0.005 sulfur, 0.013 nickel, 0.008 chromium, 0.033 aluminum, 0.015 vanadium, 0.002 titanium, 0.023 copper, and the rest is iron. CS sheets are used in weight loss tests, while CS rods are used in electrochemical tests.

### 2.3 Weight loss (WL) tests

CS sheets of 51 mm × 32 mm × 5.4 mm dimensions were polished with emery paper (of grades ranging from 320 to 1400) until a smooth surface was obtained, and then they were washed twice with distilled water. WL tests were achieved as explained earlier.^13,17^ The experiments were performed in triplicate and the experiments were repeated at different temperatures ranging from 20 to 80 °C.

### 2.4 Electrochemical tests

Potentiodynamic polarization (PDP) and electrochemical impedance spectroscopy (EIS) assessments were accomplished employing a conventional three-electrode cell with a platinum counter electrode (CE), a saturated calomel electrode (SCE) as the reference electrode and a CS rod as the working electrode (WE). The CS electrode was immersed in the test solution, until a constant potential was obtained. A “Voltalab 40”/PGZ-301 “potentiostat” was used to verify PDP curves, and a tiny personal computer with the Voltamaster-4 application was used at 293 K. PDP curves (Tafel plots) were recorded by changing the electrode potential automatically with a scan rate of 0.5 mV s^−1^ from a low potential of −800 to −300 mV with respect to the open circuit potential (OCP). All the potentials were measured against SCE. Corrosion parameters were calculated by the Tafel extrapolation method. Each experiment in this work was repeated three times to ensure reproducibility.

EIS tests were performed as described above.^20^ A slight alternating voltage perturbation (5 mV) was applied to the cell at a frequency interval of 100 kHz to 10 MHz at 293 K. Statistical analysis was carried out using the Statistical Product and Service Solutions (SPSS) software.

### 2.5 DFT and MDS details

In this work, we tried to understand the interaction of novel cationic Gemini surfactants with Fe by the DFT method in the aqueous phase.^22^ In addition, this theoretical part was carried out in order to correlate the experimentally calculated inhibitory efficacy for the cationic surfactant with the electronic reactivity.^23^ The molecular structure of the synthesized molecule was optimized in several steps until a total stability was expected in the final geometrical form, and this calculation procedure was done exploiting the Gaussian 09 software at the DFT/B3LYP scale (6-31G(d,p) basis sets).^24^

The quantum indices are *E*_LUMO_, *E*_HOMO_, Δ*E*_gap_ (eqn (1)), *μ*-polar moment, *χ*-electronegativity (eqn (2)), *η*-global hardness (eqn (3)), and Δ*N*_110_-electrons transferred degree (eqn (4)).^25^

1 Δ*E*_gap_ = *E*_LUMO_ − *E*_HOMO_

2
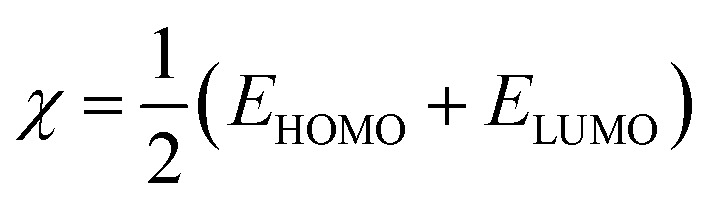
3
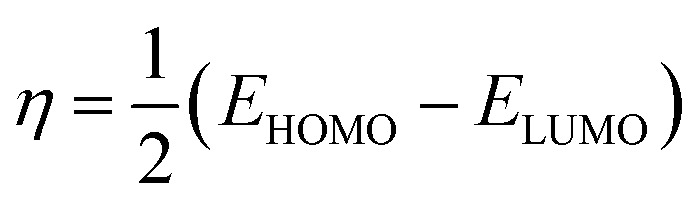
4
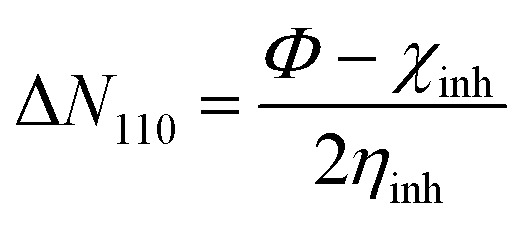


The adsorbed cationic Gemini surfactant onto the Fe(110) surface was realized by the molecular dynamics simulation (MDS) in a Forcite module recorded using the Mater. Studio^2016^ software.^26,27^ The interactions of the cationic surfactant with the Fe(110) plane were evaluated exploiting a simulation box (27.30 × 27.30 × 37.13 Å^3^) under recurring boundary conditions. The surface structure of Fe(110) plane was illustrated by a 6-layer slab model, each layer corresponding to a unit cell (11 × 11). In this simulation, we emptied the simulation supercell with 20.13 Å^3^. This is filled with 500 H_2_O, 5 H_3_O^+^, 5 Cl^−^ and the cationic surfactant. The simulated system, with a temperature of 293 K, was operated using the Andersen thermostat and an NVT ensemble at a simulation time of 600 ps and a time step of 1.0 fs, all in the COMPASS force field.^28^

## 3 Results and discussions

### 3.1 Chemical structure confirmation of CGS

#### 3.1.1 FTIR-spectra exploitation

**Fig. 1:**
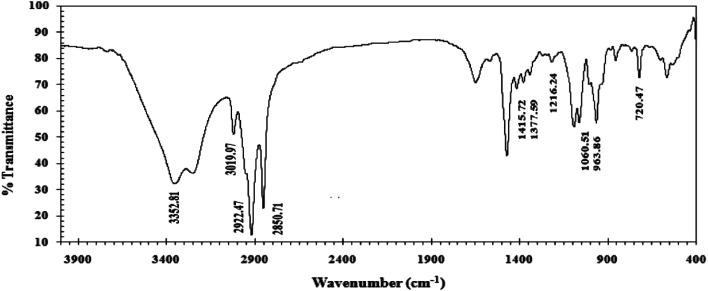
FTIR spectra of the CGS molecule.

The usual bands for alkyl fraction were at 2922.47 and 2850.71 cm^−1^ for asymmetric and symmetric stretching (CH), respectively, according to the FTIR-spectrum. They were noted at 1377.59 cm^−1^ for CH_3_, 1415.72 cm^−1^ for CH_2_, and 720.47 cm^−1^ for –(CH_2_)_*n*_– rock. The C–O stretching band at 1216.24 cm^−1^, C–N^+^ at 1060.51 cm^−1^, P–O–C at 963.86 cm^−1^ and 335.81 cm^−1^ was owing to stretching OH. The FTIR spectrum shows the predictable functional groups in the molecule, as shown in Fig. 1.

#### 3.1.2 ^1^H NMR-spectra exploitation

**Fig. 2:**
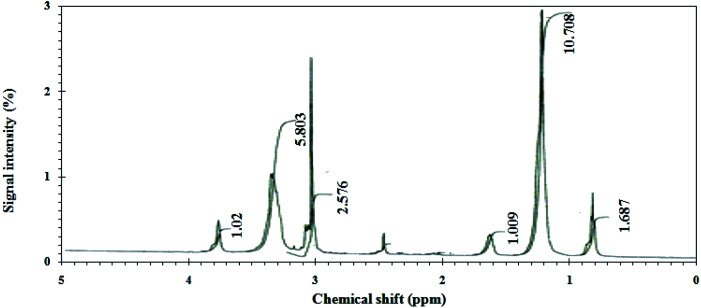
^1^HNMR spectra of the CGS molecule.

The ^1^H NMR-spectrum of the CGS-molecule show some bands at *δ* = 0.78–0.83/ppm^−1^ (t, 6H, NCH_2_CH_2_(CH_2_)_*n*_CH_3_); *δ* = 1.21/ppm^−1^ (m, 36H, NCH_2_CH_2_(CH_2_)_*n*_CH_3_); *δ* = 1.6282/ppm^−1^ (m, 4H, NCH_2_CH_2_(CH_2_)_*n*_CH_3_); *δ* = 3.04–3.12/ppm^−1^ (m, 12H, POCH_2_CH_2_N); *δ* = 3.31–3.36/ppm^−1^ (m, 34H, POCH_2_CH_2_O); *δ* = 3.78/ppm^−1^ (s, 1H, POCH_2_CH_2_OH), as illustrated in Fig. 2.

#### 3.1.3 ^31^PNMR-spectra exploitation

**Fig. 3:**
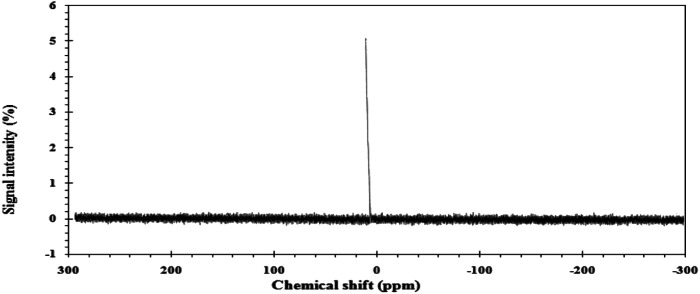
^31^PNMR spectra of the CGS molecule.

The ^31^PNMR (DMSO-d_6_) spectrum of compounds displayed *δ*_p_ signal at 0.3768/ppm^−1^, as shown in Fig. 3.

### 3.2 WL measurement

The dissolution rate (*k*), the amount of surface coverage (*θ*) and the anticorrosion efficiencies (*η*_w_) of CS in blank 1 M HCl and with CGS-molecule were calculated according to eqn (5)–(7):^19,20^5
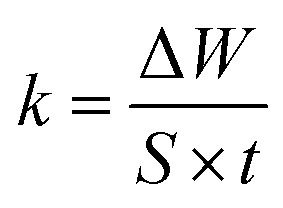
6
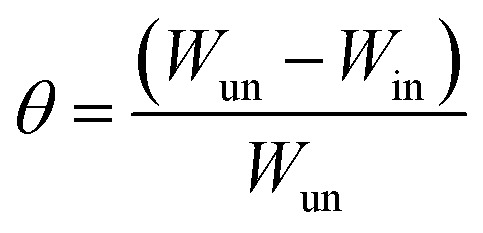
7
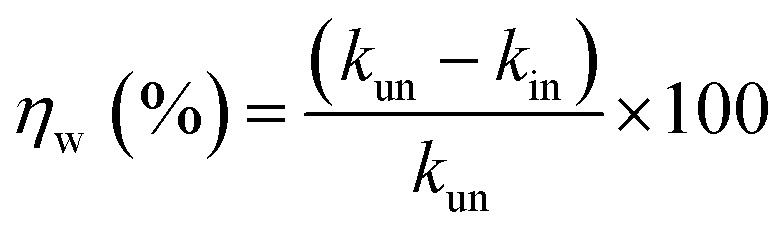
where *k*_un_ and *k*_in_ represent the corrosion rates in the 1 M HCl solution alone and with the CGS-molecule, Δ*W* is the mean WL of three parallel CS-coupons, *W*_un_ and *W*_in_ is the mass loss of CS in both the blank 1 M HCl and with the CGS-molecule, *S* is the CS-surface area in cm^2^, and *t* is the immersion time.

#### 3.2.1 Effect of CGS concentration

WL parameters of CS in blank 1 M HCl and with different concentrations of CGS are registered in Table 1. This surfactant efficiently prevents the corrosion of CS in solutions of 1 M HCl solutions owing to the occurrence of hetero atoms like N, O and P atoms. The strength of inhibition is ascribed to the nature and manner of adsorption of the CGS molecule onto the surface of the steel. WL tests revealed that the anticorrosion efficacy of CGS increases with the increase in concentration.^19^

**Table 1:** Corrosion parameters acquired from WL tests for CS in free 1 M HCl and with CGS molecule

Conc. of inhibitor (M)	20 °C			40 °C			60 °C			80 °C		
	*k* (mg cm^−2^ h^−1^)	*θ*	*η*_w_ (%)	*k* (mg cm^−2^ h^−1^)	*θ*	*η*_w_ (%)	*k* (mg cm^−2^ h^−1^)	*θ*	*η*_w_ (%)	*k* (mg cm^−2^ h^−1^)	*θ*	*η*_w_ (%)
0.00	0.4192	—	—	1.6996	—	—	5.7325	—	—	16.0965	—	—
5 × 10^−5^	0.0486	0.88	88.4	0.1954	0.89	88.5	0.6707	0.88	88.3	1.8511	0.89	88.5
1 × 10^−4^	0.0420	0.90	90.0	0.1649	0.90	90.3	0.5675	0.90	90.1	1.5775	0.90	90.2
5 × 10^−4^	0.0289	0.93	93.1	0.1139	0.93	93.3	0.3801	0.93	93.4	1.0302	0.94	93.6
1 × 10^−3^	0.0249	0.94	94.0	0.0952	0.94	94.4	0.3130	0.95	94.5	0.8837	0.95	94.5
5 × 10^−3^	0.0171	0.96	95.9	0.0646	0.96	96.2	0.2144	0.96	96.3	0.5924	0.96	96.3

#### 3.2.2 Influence of temperature

**Fig. 4:**
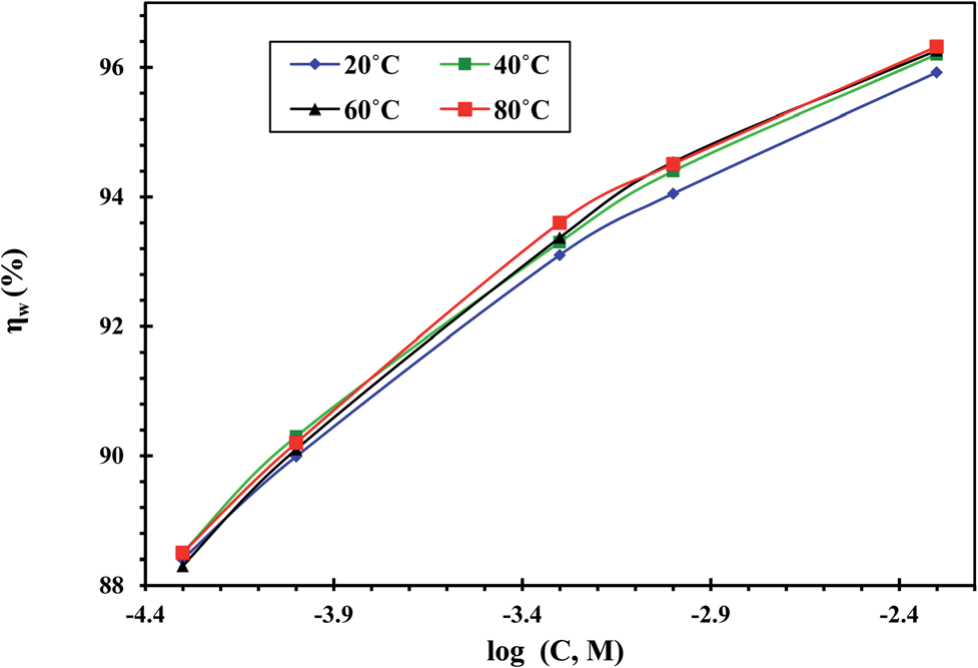
Impact of temperature on *η*_w_ obtained by WL tests for CS in free 1 M HCl and with different concentrations of the CGS molecule.

Fig. 4 displays the influence of temperature on *η*_w_ obtained from the WL tests for CS in the free acid and with different concentrations of the CGS-molecule in the temperature range of 293–353 K. The results of the weight losses and corrosion rates at different temperatures are recorded in Table 1. These outcomes demonstrate that the gradual increase in the temperature raises the corrosion rate of the CS-surfaces.^29^

Moreover, the impact of the inhibition efficacy is almost steady with the increase in temperature. The modest variation in *η*_w_ with the temperature could be due to increased CGS molecule adsorption onto the surface of the metal substrate CS.^30^ Therefore, the CS-surface is efficaciously inhibited from the corrosive medium by the anticorrosive adsorption layer.^31^

#### 3.2.3 Kinetics parameters

The activation energy (*E*_a_) values were determined from the Arrhenius relation^32,33^ as follows:8
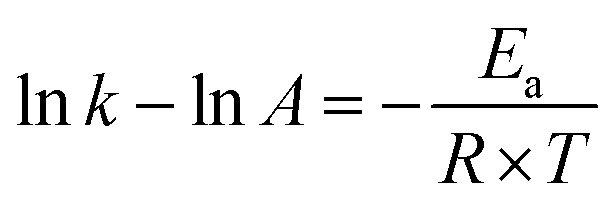
where *k* is the corrosion rate of the CS, *A* is the frequency index and *R* is the universal gas constant.

**Fig. 5:**
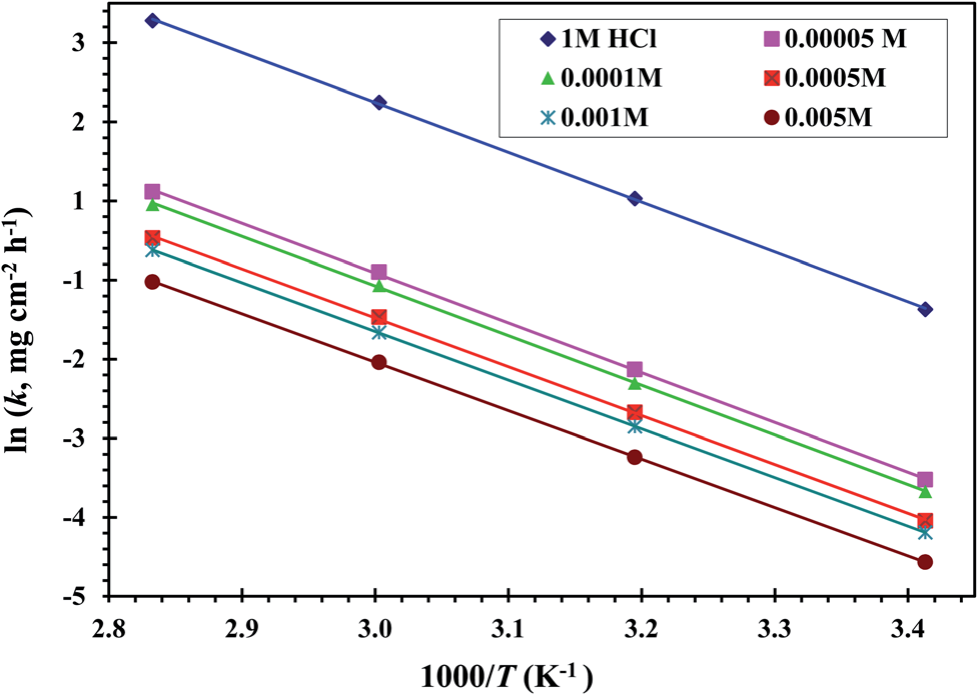
Relation between ln *k* and 1000/*T* curves for CS in 1 M HCl solution and with different concentrations of the CGS molecule.

Fig. 5 displays the relationship between ln *k* against 1000/*T* of a CS-electrode in blank 1 M HCl solution and with different concentrations of the examined CGS. Straight lines are gained with a slope equal to (−*E*_a_/*R*). The *E*_a_ values were calculated, and the results are presented in Table 2. It should be noted that in the presence of the CGS-molecule, the *E*_a_ values are lower or slightly modified when compared to free 1 M HCl. This is due to its chemical adsorption on the surface of the CS, while the opposite occurs in the case of physical adsorption.^13^

**Table 2:** Activation parameters values for CS in 1 M HCl and with certain concentrations of the CGS

Conc. of inhibitor (M)	*E*_a_ (kJ mol^−1^)	ΔH* (kJ mol^−1^)	ΔS* (J mol^−1^ K^−1^)
0.00	52.37	49.70	−156.08
5 × 10^−5^	52.33	49.67	−269.38
1 × 10^−4^	52.24	49.50	−281.00
5 × 10^−4^	51.31	48.69	−317.38
1 × 10^−3^	51.28	48.52	−329.50
5 × 10^−3^	50.93	48.27	−354.48

The following equation was used to calculate the activation enthalpy (ΔH*) and entropy (ΔS*)^32,33^ as follows:9
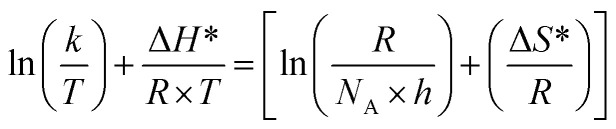
where *h* and *N* are the Planck constant and Avogadro's number, respectively.

**Fig. 6:**
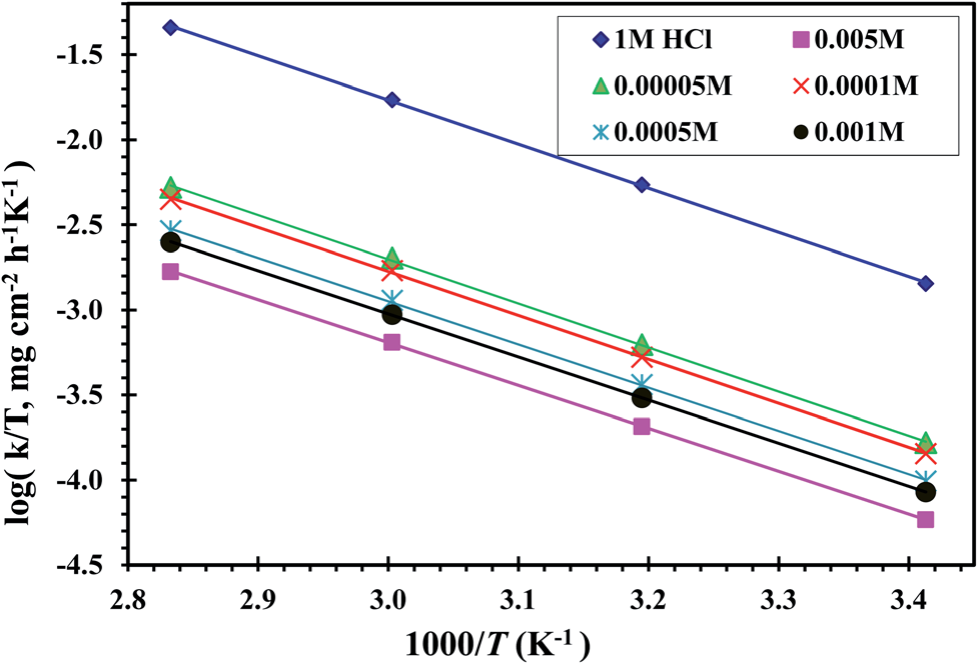
Relation between log *k*/*T* and 1000/*T* of CS in 1 M HCl solution and with different concentrations of the CGS molecule.

Fig. 6 displays a plots of ln(*k* × *T*^−1^) *vs.* (1000/*T*) for CS dissolution in free 1 M HCl solution and 0.00005, 0.0001, 0.0005, 0.0001 and 0.0005 M of the CGS-molecule. Straight lines with a slope of (−ΔH*/*R*) and an intercept of ln[(*R* × *N*^−1^ × *h*^−1^) + ΔS* × *R*^−1^] were obtained. Table 2 shows the values of the two parameters ΔH* and ΔS*. The values obtained from ΔH* have a positive sign in both uninhibited and inhibitory solutions, demonstrating the endothermic behavior of the CS corrosion. The values of ΔS* in the blank 1 M HCl solution and also in the occurrence of the CGS are negative (Table 2). This confirms that the activated complex in the rate determination step depicts an amalgam at ions instead of disengagement, which means that a reduction disruption occurs upon the transition from reactants to the activated complex. The values of ΔS* moved to more negative values when the concentration of CGS increased, showing a more stable behavior resulting in an increase in the anticorrosion efficacy.

### 3.3 Adsorption isotherm

The first step in the inhibitory effect of the CGS molecule towards the corrosion of CS in 1 M HCl solution is mainly due to its adsorption on the CS surface through the active centers in the chemical structure of the inhibitor. Thus, corrosion reactions are reduced, which occur in regions devoid of the presence of CGS.

**Fig. 7:**
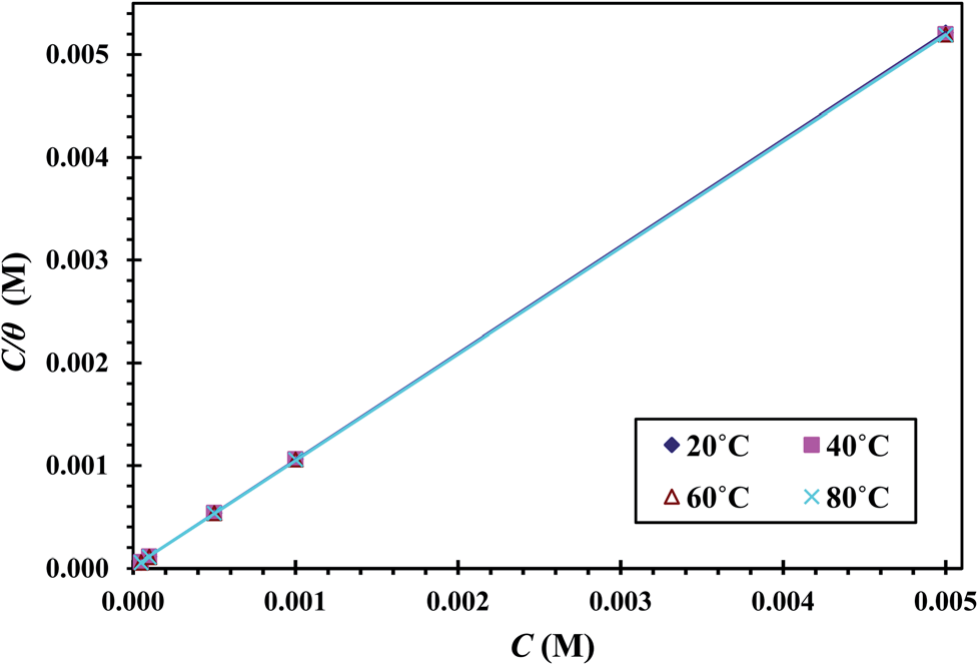
Langmuir isotherm adsorption model at different temperatures.

The values of *θ* are set in various isotherms to assign a handy isotherm, and we select the appropriate isotherm. The values of *θ* are highly valuable when verifying the adsorption properties. According to the following equation, the Langmuir isotherm is adequate for the adsorption of CGS-molecule on the surface of CS in this investigation:10
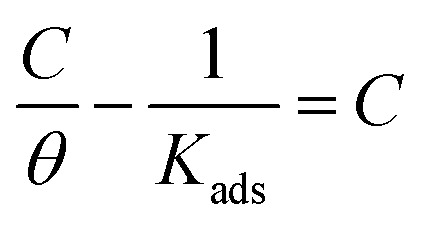
where *C* is the CGS concentration and *K*_ads_ is the equilibrium constant of adsorption. Fig. 7 displays the relationship of 
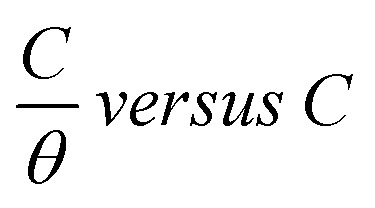
. A straight line with a slope of one unit was obtained, confirming that the Langmuir isotherm is acceptable for the adsorption process. This isotherm postulates monolayer adsorption, and there are no interaction forces between the adsorbed CGS on the CS surface.

The values of *K*_ads_ were determined from the intercept of the straight lines of the Langmuir diagram, the results are registered in Table 3. From the data in Table 3, it is obvious that the higher value of *K*_ads_ demonstrates that the CGS-molecule is vigorously adsorbed onto the surface of CS. The free energy of adsorption (Δ*G*^0^_ads_) was computed from the values of *K*_ads_ at different temperatures utilizing the next equation:

11 Δ*G*^0^_ads_ = −*R* × *T* × ln(55.5 × *K*_ads_)

where 55.5 is the concentricity of water in the solution.

**Table 3:** Thermodynamic adsorption parameters

Temperature (°C)	*K*_ads_ (×10^−4^ M^−1^)	Δ*G*^0^_ads_ (kJ mol^−1^)	Δ*H*^0^_ads_ (kJ mol^−1^)	Δ*S*^0^_ads_ (J mol^−1^ K^−1^)
20	8.22	−37.35	0.33	128.61
40	8.29	−39.92		128.61
60	8.35	−42.50		128.61
80	8.41	−45.07		128.61

Table 3 presents the thermodynamic values for the adsorption of the CGS-molecule in 1 M HCl solution on the CS-surface at different temperatures. The negative signs of Δ*G*^0^_ads_ emphasize that the adsorption of the CGS molecule on the CS surface is stable and spontaneous.

The adsorption enthalpy could be computed using the Van't Hoff equation:^34^12
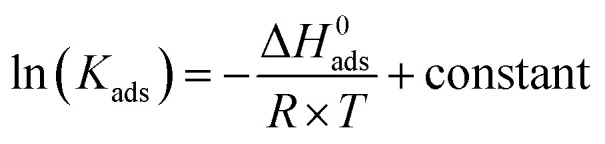
where Δ*H*^0^_ads_is the enthalpy of adsorption.

**Fig. 8:**
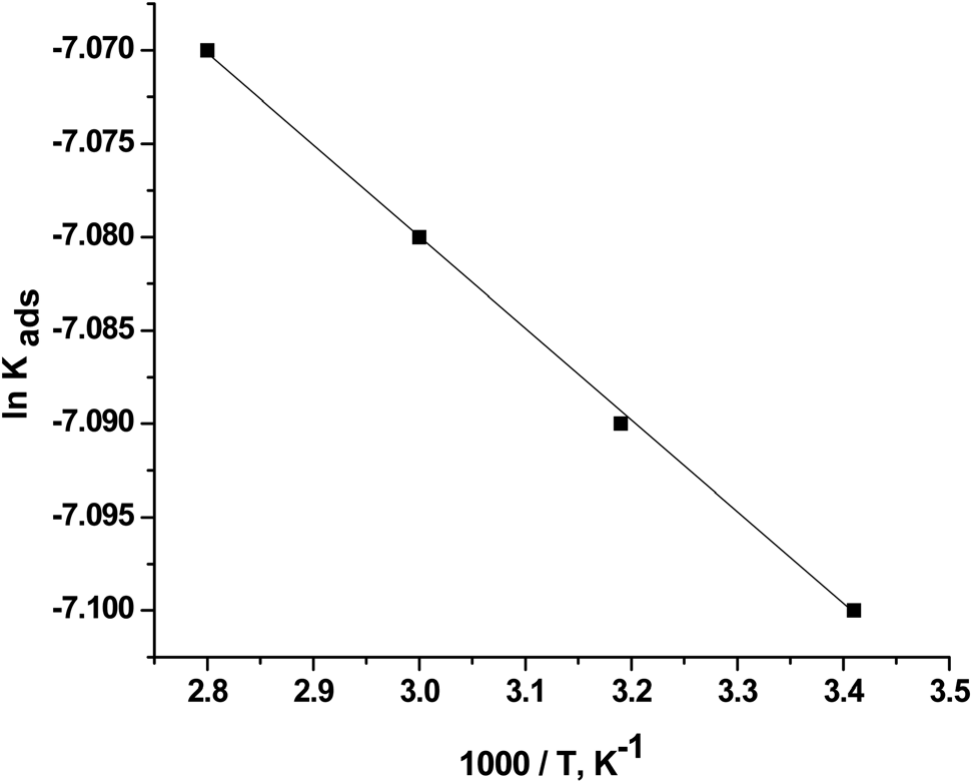
Plots between ln *k*_ads_ and 1000/*T* of CS in 1 M HCl and with different concentrations of CGS.

Fig. 8 shows the diagram between ln *K*_ads_ and 1000/*T*. A linear regression with a slope of (−Δ*H*^0^_ads_/*R*) was obtained. The Δ*H*^0^_ads_ values were calculated, and the results are presented in Table 3. The positive Δ*H*^0^_ads_ value indicates that CGS adsorption onto the CS surface is endothermic.

The entropy of adsorption (Δ*S*^0^_ads_) can be determined by applying the next equation and inserted in Table 3:

13 Δ*G*^0^_ads_ = Δ*H*^0^_ads_ − *T* × Δ*S*^0^_ads_

The positive sign of Δ*S*^0^_ads_ is also linked to the replacement process, which could be assigned to the enhanced entropy of the solvent and a stronger positive entropy of desorption of water.^35^ A greater disturbance resulting from a larger number of water molecules is another explanation. It could be desorbed from the CS-surface by a single CGS-molecule.^36,37^

### 3.4 PDP results

**Fig. 9:**
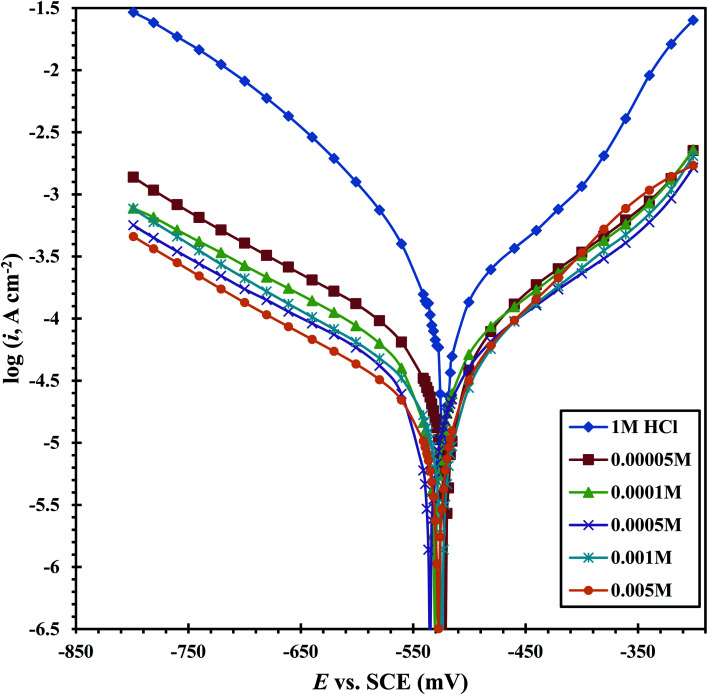
PDP curves for CS in 1 M HCl alone as well as containing different concentrations of CGS.

Fig. 9 shows the PDP tests of the CS-electrode in 1 M HCl solution alone and with different concentrations of the CGS molecule. The acquired corrosion data, *e.g.* anodic (*β*_a_) and cathodic (*β*_c_) Tafel slopes, corrosion potential *E*_corr_, corrosion current density *i*_corr_ and anticorrosion efficacy *η*_PDP_ are presented in Table 4. The anticorrosion efficacy is computed as follows:14
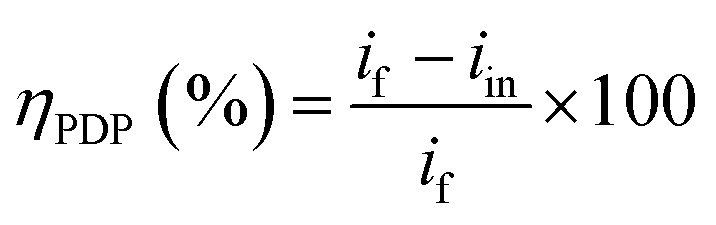
where *i*_f_ and *i*_in_ are the corrosion current densities in the absence and presence of different concentrations of the CGS-molecule, respectively.

**Table 4:** Corrosion parameters obtained from the PDP tests of CS in 1 M HCl and with certain concentrations of the CGS molecules^a^

Conc. of inhibitor (M)	*E*_corr_ mV (SCE)	*i*_corr_ (mA cm^−2^)	*β*_a_ (mV dec^−1^)	*β*_c_ (mV dec^−1^)	*η*_p_ (%)
0.00	−524.0	0.4245 ± 0.0054	166.2	−148.1	—
5 × 10^−5^	−521.5	0.0485 ± 0.0014	145.4	−195.3	88.57
1 × 10^−4^	−530.5	0.0420 ± 0.0013	151.3	−214.6	90.11
5 × 10^−4^	−535.0	0.0306 ± 0.0011	150.7	−216.4	92.79
1 × 10^−3^	−524.5	0.0257 ± 0.0013	129.4	−192.3	93.94
5 × 10^−3^	−527.5	0.0184 ± 0.0007	102.0	−202.5	95.66

a *i*_corr_ = mean ± 
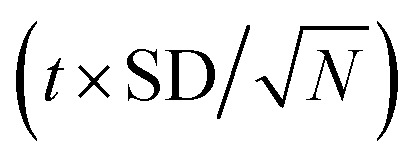
 where *t* = 2.776 at confidence limit 95%, SD = standard deviation, *N* = no of measurements = 5.

It is obvious from Fig. 9 that a transition region was initially obtained, where the current slowly changes to a positive or negative direction (curves characterized anodic and cathodic polarization). After this region, the potential increases rapidly with the current (the Tafel region). The transition region^38^ starts from the free corrosion potential and expands up to the onset of the Tafel region, due to the simultaneous cathodic H2 evolution and anodic dissolution of the CS. Table 4 clearly shows that after the addition of the CGS-molecule, we find that the change in the values *β*_a_ and *β*_c_ is low when compared to the blank solution. Moreover, the *E*_corr_ values remain constant and shift in *E*_corr_ (≤11 mV). Previous studies reported that an inhibitor is considered as a mixed inhibitor if the *E*_corr_ value of the inhibitor including the solution is ±85 mV or more than the value of the blank acidic solution. This confirms that the examined CGS-molecule is classified as an anti-corrosion of the mixed type.^39^ It appears that this CGS-molecule is simply adsorbed onto the CS-surface by covering the active sites, with no effect on the anodic and cathodic reaction mechanisms.^38^ This can be explained by the CGS-molecule adsorption onto the CS-surface. In addition, it can be observed from Table 4 that the *i*_corr_ values decrease, and hence the corrosion rate is reduced, while the anticorrosion efficacy enhances with the augmentation in the CGS concentration. These outcomes confirmed the effectiveness of inhibition of the CGS-molecule.

### 3.5 EIS results

**Fig. 10:**
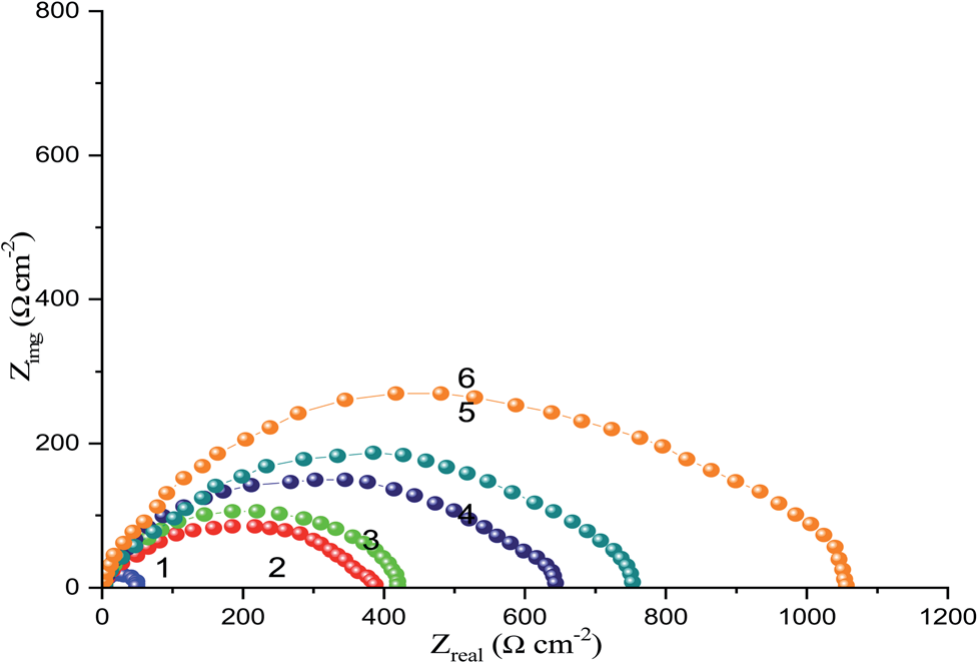
Nyquist diagrams for CS in free 1 M HCl and with different concentrations of the CGS molecule (1) 1 M HCl, (2) 0.00005 M, (3) 0.0001 M, (4) 0.0005 M, (5) 0.001 M and (6) 0.005 M.

Fig. 10 shows the Nyquist plots of the CS-electrode in 1 M HCl solution with and without different concentrations of the CGS molecule. A large capacitive loop was observed. The shape of the capacitive loops demonstrates that the charge-transfer controls the CS corrosion. Obviously, as the concentration of the CGS-molecule increases, the Nyquist plots are greatly altered and the diameter of the capacitive loop increases. This signifies that the anticorrosion efficacy is proportional to the concentration of the CGS-molecule. All Nyquist diagrams were composed of a “low” semicircle with one capacitive ring and one depressed semicircle. This behavior is advantageous for solid electrodes and is often called frequency dispersal, which is due to the coarseness and heterogeneity of the hard surface.^40,41^

**Fig. 11:**
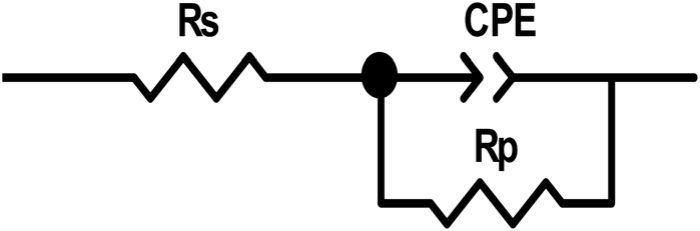
Equivalent circuit exploited for EIS tests.

Nyquist diagrams were analyses by the appropriateness of the experimental outcomes to a parabolic circuit, as shown in Fig. 11, which contains the solution resistance *R*_s_ and the constant phase element (CPE) that is set parallel to polarization resistance element, *R*_p_. Often a CPE is utilized in a model in place of a capacitor to compensate for heterogeneity in the system. Stellar compatibility with this model was acquired for all experimental outcomes. The CPE was recognized by two values, *Q* and *n*, and was determined using the following equation:^41^

15 *Z*_CPE_ = *Q*^−1^ × (*i* × *ω*_max_)^−*n*^

where *Q* is the CPE constant, *ω*_max_ is the angular frequency, *ω* = 2π*f*_max_ (where *f* is the frequency at which the imaginary component of the resistance reaches its highest), *i* is the imaginary number and *n* is the CPE exponent, which can be used to determine surface unevenness or roughness.^33,34^ When *n* = 1, the CPE behaves like an ideal double-layer capacitance (*C*_dl_).^42^

**Fig. 12:**
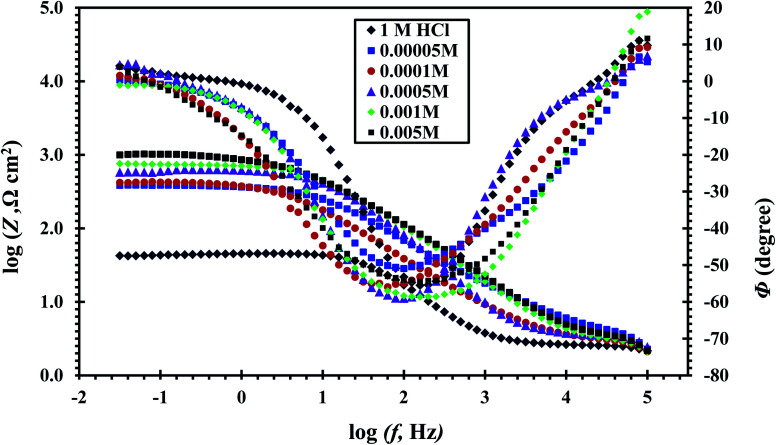
Bode diagrams of CS in 1 M HCl and with different concentrations of the CGS molecule.

The value of *n* decreases indicating the reduction of surface homogeneity. The Bode and phase angle plots of the CS-electrode in free 1 M HCl and with CGS-molecule are shown in Fig. 12, which shows that as the adsorption of CGS-molecule on the CS-surface increases in 1 M HCl solution, the increase in absolute impedance at lower frequencies in the Bode diagrams clarifies more protection.

Table 5 represents the impedance data acquired from EIS tests in a free 1 M HCl solution and with augment concentrations of the CGS-molecule employing the equivalent circuit model. The same table shows the computed double-layer capacitance values (*C*_dl_), which were determined using the following equation:

16 *C*_dl_ = *Q* × (*ω*_max_)^−*n*^

**Table 5:** EIS parameters for corrosion of CS in free 1 M HCl and with certain concentrations of the CGS^a^

Conc. of inhibitor (M)	*R*_s_ (Ω cm^2^)	*Q* (Ω^−1^ s^*n*^ cm^−2^)	*n*	*R*_p_ (Ω cm^2^)	*C*_dl_ (μF cm^−2^)	*η*_*z*_ (%)
0.00	2.10	0.1605	0.91	48.4780 ± 1.0964	102.2	—
5 × 10^−5^	2.27	0.0186	0.69	402.4000 ± 4.1732	37.4	87.94
1 × 10^−4^	2.11	0.0174	0.73	429.0000 ± 3.1651	83.3	88.76
5 × 10^−4^	2.45	0.0104	0.88	644.2000 ± 3.4449	25.1	92.50
1 × 10^−3^	2.15	0.0090	0.80	756.6000 ± 2.8581	24.1	93.64
5 × 10^−3^	2.60	0.0071	0.86	1066.4000 ± 6.3667	30.7	95.47

a *R*_p_ = mean ± 
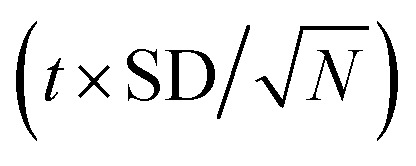
 where *t* = 2.776 at confidence limit 95%, SD = standard deviation, *N* = no of measurements = 5.

The *R*_p_ values were used to calculate the anticorrosion efficacy (*η*_*z*_), due to the following equation:17
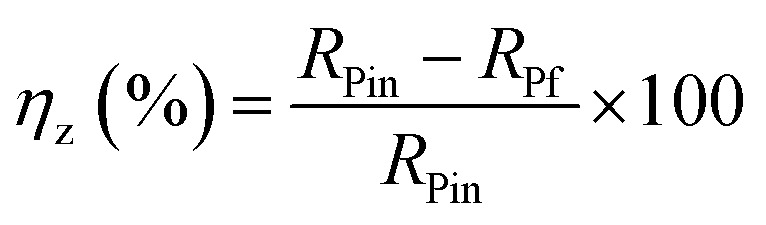
where *R*_pf_ and *R*_pin_ are the polarization resistance in the devoid of and with the CGS-molecule. The outcomes obtained are presented in Table 5. The exchange of water molecules and other ions that were primarily adsorbed onto the surface of the CS by the CGS-molecule, forming an adherent layer, which diminishes the number of active sites required for the dissolution process and causes the *R*_p_ value to rise with the CGS concentration, resulting in an increase in the anticorrosion effectiveness. Due to the formation of an adsorbed layer on the CS-surface, the addition of a CGS-molecule decreases *C*_dl_.^43^ Obviously, the shapes of the diagrams of inhibiting electrodes are not fundamentally variant from those of non-inhibited electrodes. The existence of the CGS-molecule raises the impedance but does not alter the behavior. These outcomes emphasized the results of PDP tests of the CGS molecule do not change the anodic reactions accountable for corrosion. It mainly retards the corrosion by adsorbing it onto the surface of CS.^44^

### 3.6 Interpretation of anticorrosion

**Fig. 13:**
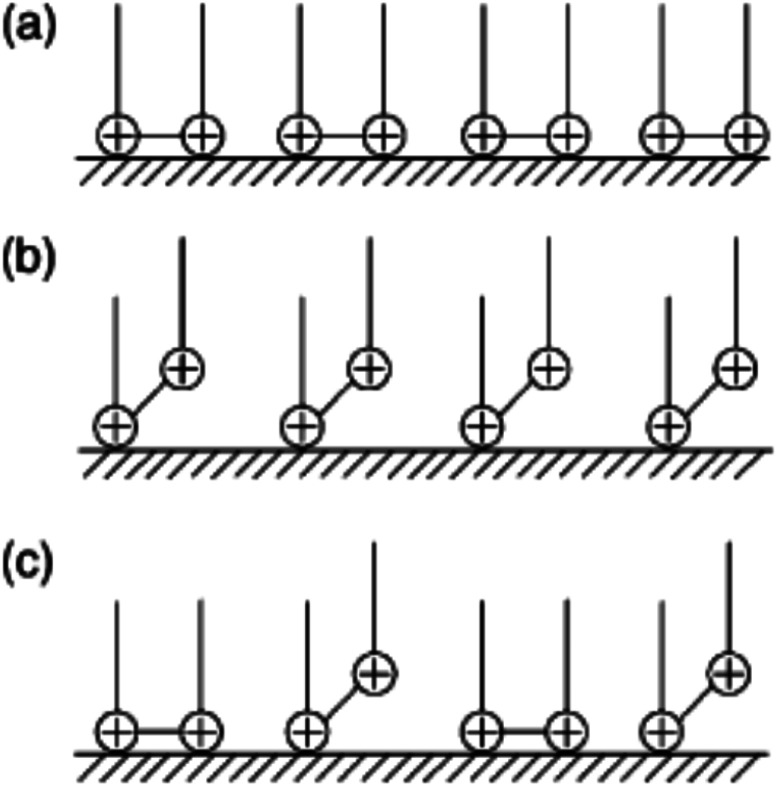
Visualization of the adsorption of CGS on the surface of CS.

The CS corrosion rate is reduced by adsorption of CGS on its surface, since the CGS-molecule exploited as an anticorrosion material has two hydrophilic and two hydrophobic groups. Multiple layers can be formed on the CS-surface at surfactant concentrations higher than the critical micelle concentration.^45^ We can come in three different scenarios for the adsorption of CGS. Before multiple layers forms, three proposals can exit: (a) two hydrophilic ionic groups of CGS are adsorbed at the CS-surface site; (b) one hydrophilic group is adsorbed at the surface site, while the other is free in the solution phase, and (c) both (a) and (b) co-exist. The vision of the three proposals is shown in Fig. 13.

**Fig. 14:**
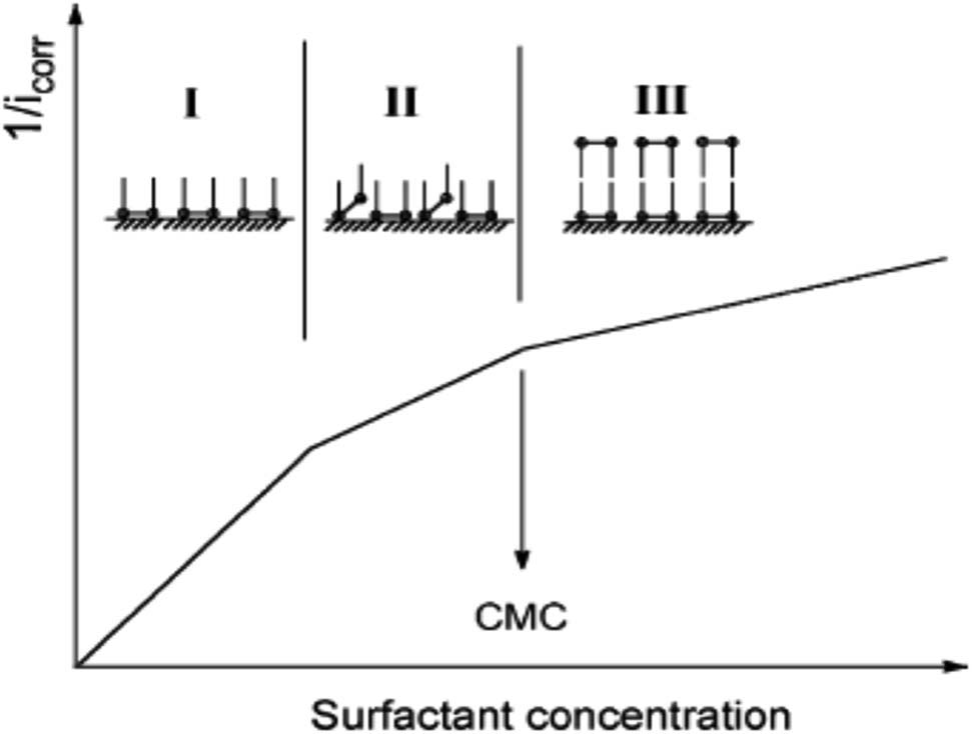
Adsorption manner of CGS on the CS surface at different concentrations.

Let us clarify more in Fig. 14: from first proposal (a), the primary adsorption mechanism should be with low concentrations of the CGS-molecule (phase I), and the CGS-molecule tend to be adsorbed onto the CS-surface at high concentrations according to proposal (b). However, in reality, proposal (c) should make more sense due to the interaction between molecules of CGS (phase II). Furthermore, the augmentation in the CGS concentration is higher than that of CMC, and multiple layers may form as in the conventional single-chain surfactant inhibitor system (phase III). It can elucidate why the straight-line slope of the relationship (*C*/*θ vs. C*) of CGS molecule, 1.043, is slightly greater than unity, that is, the apparent coverage inferred direct WL tests are smaller than the true coverage. Above this concentration, a further augmentation in the concentration of the CGS-molecule leads to the gradual formation of multiple layers, just as it is in the anti-corrosion system exploiting conventional surfactants. The CGS-molecule is known to be adsorbed on the negatively charged steel surface in competition with the H^+^ ion in order to limit the H_2_ evolution reaction. Moreover, the chloride ions are capable of enhancing the adsorption of the organic cations in the solution by forming the intermediate bridges between the steel surface and the positive end of the CGS-molecule. Besides the physical adsorption, the CGS-molecule can be adsorbed onto the CS-surface *via* the chemisorption mechanism, including the replacement of water molecules from the CS-surface, donor–acceptor interactions between heteroatoms, lone pair of electrons on N, O and P atoms and the vacant d-orbitals of the surface iron atoms, so that we can describe these synthesized CGS-molecules as mixed-type inhibitors. The value of thermodynamic parameters indicates that the endothermic adsorption process of the CGS-molecule mainly enhances chemisorption. Unchanged or lower values of activation energy compared to the blank confirmed the chemisorption mechanism.

### 3.7 DFT reactivity

**Fig. 15:**
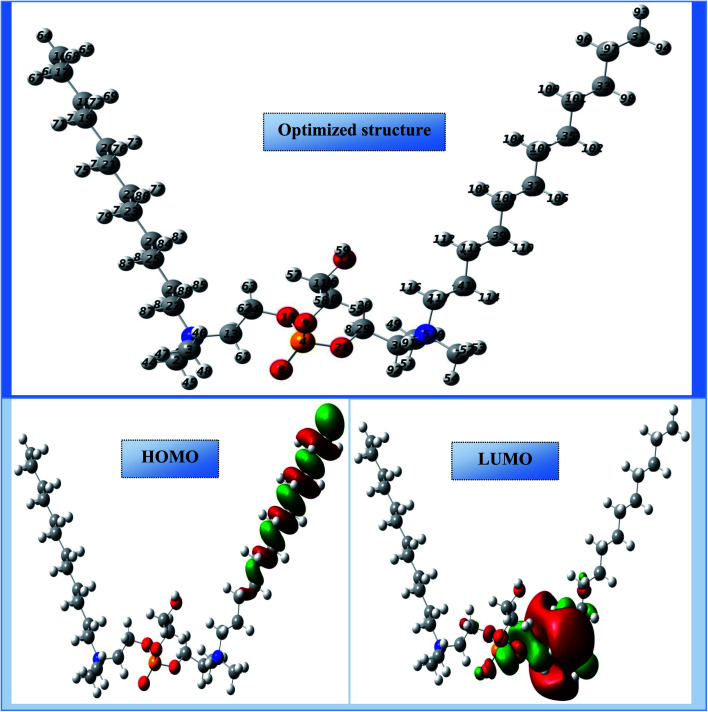
Optimized skeleton and HOMO/LUMO distributions of CGS.

The electron density distributions on the molecular skeleton in an organic molecule can be exploited to determine the regions of reactivity and the active sites in these regions.^46^ Using frontier molecular orbitals (FMO), *i.e.* the highest occupied molecular orbitals (HOMO) and the lowest unoccupied molecular orbitals (LUMO), we can identify and analyze this distribution.^47^ The molecular structure portrayed in Fig. 15 indicates a more stable spatial geometry with non-imaginary frequencies. The onset of this spatial aspect may be due to the existence of groups attached to the motif such as (hydroxy-octaoxahexacosyl-oxy-phosphoryl-oxy-ethyl)-*N*,*N*-dimethyldodecan-1-aminium. Fig. 15 also illustrates the occupied (HOMO) and unoccupied (LUMO) parts of the electron density in cationic Gemini surfactants. It is well observed that the LUMO and HOMO electronic densities appeared on the half of the synthesized compound, and this behavior may be due to the presence of a symmetry plane. Therefore, the HOMO density is positioned on one of the carbon chains (C_12_H_25_–), while the LUMO density is distributed on the hydroxy-octaoxahexacosyl)oxy)phosphoryl)oxy)ethyl)-*N*,*N*-dimethyldodecan-1-aminium part. These parts of the tested compound can react with the CS surface to reduce the degradation phenomenon of this steel.

The main quantum electro-level descriptors assigned to the cationic form are recorded in Table 6. As shown in this table, the occupied level HOMO (−10.590 eV) and the unoccupied level LUMO (−5.251 eV) exhibit the highest capacity of the synthesized compound to donate/accept electrons. Consequently, this can lead to a high tendency to adsorb on to the surface of CS.^48,49^ The chemical reactivity of the cationic Gemini surfactant was also estimated and well sustained by the gap energy (Δ*E*_gap_) in this case equal to 5.339 eV. This low value of Δ*E*_gap_ indicates a higher anticorrosion reactivity.^50^ The high chemical reactivity of the cationic surfactant is also supported and validated by the high polar moment value (29.320 D). Furthermore, the high inhibition efficiency of the cationic surfactant may be due to the strong donor–acceptor interactions with iron atoms, and this property can be justified by the low values of the *χ* (7.920 eV) and *η* (2.669 eV) descriptors.^51^ The power of electron donation by the cationic form was estimated *via* the fraction of the emitted electron number Δ*N*_110_, and this descriptor helps globally to understand the relational nature of the inhibitor molecule and the adsorption surface.^52^ The computational results suggest that the value of this descriptor is more negative (−0.581), indicating an electron acceptor status of the optimized compound. The reason for this may be the cationic form of the surfactant, which is charged at the nitrogen atoms. An inhibitory efficacy of an organic molecule can be assessed and analyzed by the presence of active centers distributed over its molecular structure. Additionally, this efficiency increases with the active sites of local reactivity. These sites can create chemical bonds with the active centers lying on the surface of the carbon steel.^53^ In this new research, the local selectivity of cationic Gemini surfactant was calculated using the Fukui functions ((*f*_*i*_^−^; electrophilic attack) and (*f*_*i*_^+^; nucleophilic attack)) with the most condensed functions present at the most active sites (atoms).^54^Table 7 groups the various local activity sites represented by the most appropriate atoms that have the highest density representing the nucleophilic (acceptor) and electrophilic (donor) electron centers. As displayed in Table 7, the molecule investigated under the cationic form shows a more outstanding attractive character. This means that there are several heteroatoms with a high *f*_*i*_^+^ density, which are able to capture and accept electrons coming from the surface of the carbon steel. The synthesized compound does not present any electron donor site; this shows that the chemical reactivity takes place in an irreversible manner. This characteristic requires the protection of the chosen steel against corrosion, indicating that the cationic Gemini surfactant is considered to be a good corrosion inhibitor.

**Table 6:** DFT-descriptors describing the reactivity of cationic form studied

Cationic Gemini surfactant	*E*_HOMO_ (eV)	*E*_LUMO_ (eV)	Δ*E*_gap_ (eV)	*χ* (eV)	*η* (eV)	Δ*N*_110_	*μ* (D)
	−10.590	−5.251	5.339	7.920	2.669	−0.581	29.320

**Table 7:** *f*_*i*_^−^and*f*_*i*_^+^for the most relevant atoms of cationic Gemini surfactant

Atoms	*f*_*i*_^−^	*f*_*i*_^+^
N (1)	0.008	0.007
C (2)	0.014	0.013
C (3)	0.007	0.006
P (4)	0.059	0.097
N (5)	0.006	0.006
C (6)	0.007	0.006
C (7)	0.014	0.013
O (8)	0.108	0.114
O (9)	0.059	0.065
C (10)	0.017	0.018
C (11)	0.015	0.014
O (12)	0.020	0.019
C (13)	0.018	0.018
C (14)	0.026	0.026
O (15)	0.051	0.056
C (16)	0.001	0.001
C (17)	0.000	0.000
C (18)	0.000	0.000
C (19)	0.000	0.000
C (20)	0.000	0.001
C (21)	0.000	0.001
C (22)	0.001	0.001
C (23)	0.001	0.001
C (24)	0.001	0.001
C (25)	0.001	0.001
C (26)	0.000	0.000
C (27)	0.001	0.001
O (28)	0.071	0.076
C (29)	0.019	0.019
C (30)	0.018	0.017
C (31)	0.001	0.001
C (32)	0.000	0.001
C (33)	0.001	0.001
C (34)	0.000	0.000
C (35)	0.001	0.001
C (36)	0.000	0.000
C (37)	0.001	0.001
C (38)	0.001	0.001
C (39)	0.002	0.002
C (40)	0.001	0.001
C (41)	0.003	0.003
C (42)	0.002	0.002

### 3.8 MDS approach

**Fig. 16:**
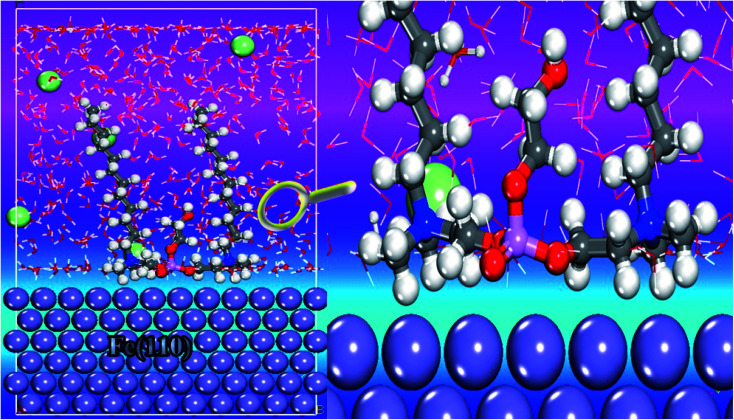
Side views of the stable adsorption configuration of the cationic Gemini surfactant onto the Fe(110) surface.

The molecular dynamics simulation (MDS) approach was carried out and the simulated system approached balance until the energy and temperature of this system were fully stable.^55^Fig. 16 illustrates the suitable adsorption configuration of the cationic Gemini surfactant on the Fe(110) surface. As a visual analysis of this image, vertical (perpendicular) adsorption was observed on the adsorption surface in which the two carbon chains (C_12_H_25_–) and the unit chain (–CH_2_–CH_2_–O–) are oriented upwards. In addition, the adsorption centers, namely, the O, N, S and P atoms, are positioned just above the surface of the contact containing iron atoms.^56^ This adsorption performance indicates that the cationic form (surfactant) acts through the active part (qor). This part can be responsible for protecting the surface of carbon steel against corrosion. In addition, this active region can receive the electrons from the occupied orbitals located in the metal surface.

The interaction energies (*E*_interaction_) of the cationic Gemini surfactant/Fe(110) are calculated as follows:^57^

18 *E*_interaction_ = *E*_total_ − *E*_surface+solution_ − *E*_binding_

where *E*_total_ is the total energy of the whole studied system, *E*_surface+solution_ is the total energy of the Fe(110) surface and solution without the inhibitor and *E*_inhibitor_ represents the energy of the free inhibitor molecule.

**Fig. 17:**
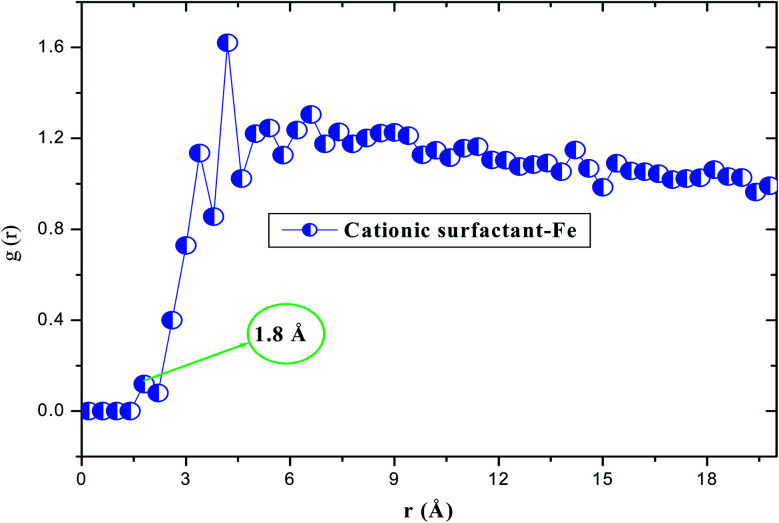
RDF of the cationic surfactant onto the Fe(110) surface at 293 K.

The interaction energy was calculated when the system was in the steady state. The calculated negative value of *E*_interaction_ (−872.213 kJ mol^−1^) reveals the adsorption spontaneity of the cationic form on the iron surface at the atomic level, moreover, the minimum energy value indicates a strong interfacial interaction (inhibitor/Fe(110)).^58^ These simulation data suggest that our compound has the best inhibiting action for carbon steel, which compares well with the experimental results. According to the nature of the adsorption, *i.e.* physical, chemical or both, we decided to use the radial distribution function (RDF) method as the frequently employed approach.^59^ For this reason, Fig. 17 depicts the insights obtained from RDF; we can see that the value of first peaks is less than 3.5 Å. This behavior indicates that the study product chemically adsorbs onto the iron surface, indicating a better protection against the corrosion process.^60^

## 4 Conclusions

(1) In a 1 M HCl solution, synthetic CGS displays effective anticorrosion against CS corrosion.

(2) The anticorrosion efficacy increases with the increase in the concentration of the CGS molecule in the temperature range of 293–353 K.

(3) The anticorrosion efficacy reached 95.66% at 5 × 10^−3^ M of the CGS molecule using PDP measurements.

(4) The PDP curves indicate that the synthesized CGS molecule is of mixed type.

(5) Activation energy did not change with the addition of inhibitor.

(6) The adsorption of the CGS-molecule onto the CS-surface follows the Langmuir isotherm.

(7) The local reactivity and distribution of the electronic density of HOMO/LUMO shows that the CGS-molecule contains highly reactive centers distributed throughout the molecular structure, which are the cause of their inhibitory properties.

(8) MD simulations reveal the strong interaction between the screened molecules and the iron surface.

## Conflicts of interest

There are no conflicts to declare.

## Supplementary Material
